# Chitosan pyrolysis in the presence of a ZnCl_2_/NaCl salts for carbons with electrocatalytic activity in oxygen reduction reaction in alkaline solutions

**DOI:** 10.1038/s41598-024-72411-1

**Published:** 2024-10-08

**Authors:** Maria K. Kochaniec, Marek Lieder

**Affiliations:** 1 Faculty of Chemistry, Warsaw University of Technology, Noakowskiego 3, 00-664 Warsaw, Poland; 2 Chemical Faculty, Department of Process Engineering and Chemical Technology, Gdansk University of Technology, Narutowicza 11/12, 80-233 Gdansk, Poland

**Keywords:** Carbonized chitosan, Sustainable energy, Nanostructures, ORR activity, Microporosity, Energy, Chemistry, Electrocatalysis

## Abstract

The one-step carbonization of low cost and abundant chitosan biopolymer in the presence of salt eutectics ZnCl_2_/NaCl results in nitrogen-doped carbon nanostructures (8.5 wt.% total nitrogen content). NaCl yields the spacious 3D structure, which allows external oxygen to easily reach the active sites for the oxygen reduction reaction (ORR) distinguished by their high onset potential and the maximum turnover frequency of 0.132 e site⁻^1^ s⁻^1^. Data show that the presence of NaCl during the synthesis exhibits the formation of pores having large specific volumes and surface (specific surface area of 1217 m^2^ g^−1^), and holds advantage by their pores characteristics such as their micro-size part, which provides a platform for mass transport distribution in three-dimensional N-doped catalysts for ORR. It holds benefit over sample pre-treated with LiCl in terms of the micropores specific volume and area, seen as their percentage rate, measured in the BET. Therefore, the average concentration of the active site on the surface is larger.

## Introduction

The renewable biomass can be chemically transformed into various energy carriers or carbonaceous solid materials for special applications, like gas sorption^1,2^ or catalysis^3,4^. The carbon possessing high specific surface area is a prerequisite for individuals obtained by simple pyrolysis of the biomass in an inert gas atmosphere^5^. Usually, a post etch treatment is necessary at high temperatures. This treatment is very effective indeed, and yields porous activated carbons, but it involves the use of highly corrosive agents like KOH^6–9^. Chitosan (CS) is a good example of a raw material for the carbon production, which needs post activation treatments^10,11^. However, controlled syntheses of the CS-based activated carbons have been also produced by a one-step thermal treatment in the presence of the etching (ZnCl_2_)^12,13^ or gas releasing (Na/K/Ca carbonates) agents^14–16^.

Another promising route opens the use of inorganic salt melts (IM)^17^. However, salts like, LiCl, NaCl, or KCl turn into a fluid reaction environment only at the very advanced stages of the pyrolysis process because they have relatively high melting points (610–800 °C)^18^. It appears to be an important point to make, since we may assume that the melt percolates through the biomass are more effective, the activation process. Fortunately, chlorides of alkali metals mixed with zinc chloride melt at much lower temperatures (eutectics), e.g. NaCl-ZnCl₂ melts at 260 °C. At the early stages of the carbonization, ZnCl₂ through dehydration action facilitates the formation of reactive double bonds, cyclo-additions and finally the creation of carbon structures^19^. The resulting carbon is a highly reducing agent, and as such it reduces zinc ions, what drives the pores formation (etching). Zinc chloride is intercalated into the carbon matrix to produce pores at temperature above the melting point of chemical agent. During the described processes the ionic melts percolates through the solid and fills up all voids, pores, crevices, spaces between carbon layers etc^20,21^. The presence of the salts in the carbonaceous structure also during a cooling stage prevents all sorts of 'empty' spots from collapsing, and even may exerts some sort of a compressive stress on the carbon surroundings^22^. As we have reported in our previous paper, the latter phenomenon resulted in appearance of curvature in the graphitic sheets^23^. These findings may support the view that alkali metals chlorides might strongly influence the shape and dimension of the carbonaceous structures created during pyrolysis, what is of great importance for their catalytic activity at nanoscale^24^. In the cited paper we presented the results of the chitosan pyrolysis assisted by a ZnCl_2_/LiCl melt. This time we would also like to report a one-pot chitosan pyrolysis, but performed in the presence of a ZnCl_2_/NaCl mixture. Some physical properties of NaCl differ from that of LiCl, what might induce other topological defects of the carbonaceous products. Firstly, atomic and ionic radii (6-coordinate) for sodium, 1.86 Ȧ and 1.02 Ȧ are larger than that for lithium, 1.52 Ȧ and 0.76 Ȧ^25^. Secondly, the melting point of NaCl (801 °C) is almost 200 °C higher than the one for LiCl (605 °C).

We decided to investigate the impact of NaCl melt (containing ZnCl_2_) on the morphology of the carbons obtained from CS. As our^23^ and others studies have shown^26–28^ these type of materials exhibit catalytic effect toward oxygen reduction reaction, because of the presence of nitrogen functionality, specific topology and morphology of the structures. The main aim of the work was the synthesis of the N-doped carbon obtained in the presence of a ZnCl_2_/NaCl salt melt pyrolysis of chitosan and their full characterization, including structural and morphological. We did not expect NaCl to boost the intrinsic activity of these catalysts through the increase in the number of highly active nitrogen groups, but we hoped that this route would yield the spacious 3D structure, which allows external oxygen to easily reach those active sites for reduction.

## Results and discussion

### Structure characterization

**Fig. 1:**
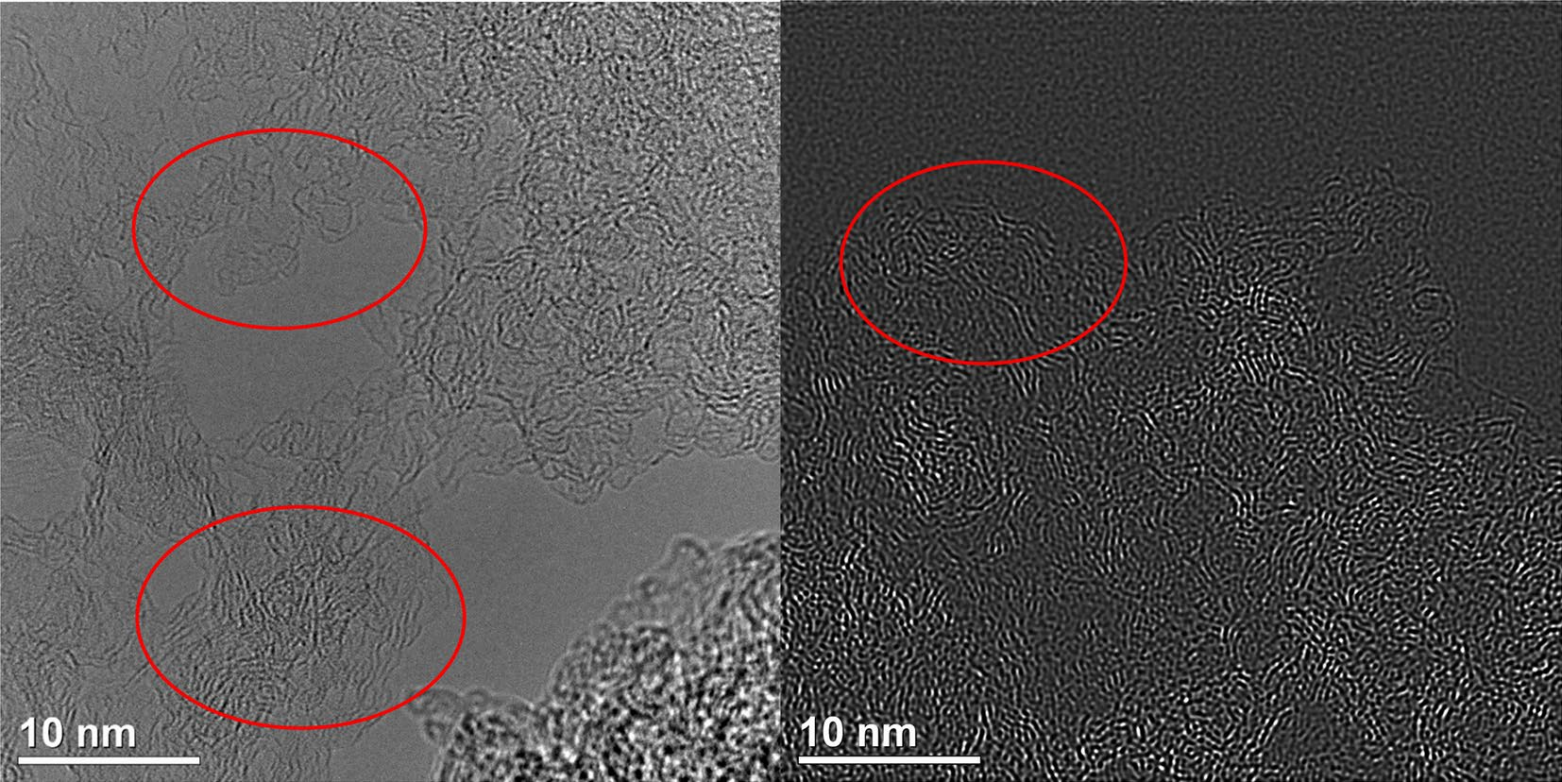
HRTEM images of the CH_NaCl sample (voids are marked by red ellipse).

Heat-treated materials obtained by plain pyrolytic degradation of CS are composed of dense domains, which have random orientation to each other and lack long-range order^23^. In contrast, the same precursor pyrolyzed in the presence of ZnCl_2_ and NaCl yields the carbonaceous chains and plains forming semi closed structures. In contrast, there are also circular and randomly arranged voids occurring in the entire volume of the samples. (Fig. 1). The presence of NaCl during pyrolysis was not only beneficial in the formation of the pores, but also contributed to enlargement. At elevated temperatures the CS polymers are converted into polycyclic aromatic compounds. Thus, we assume, the layered and wrinkled strings observed in the HRTEM images are essentially aromatic molecules that create this specific topology. The SEM (Fig. S1) and particularly HRTEM images show carbon flakes and sheets characterized by percolation structures, although visible distorted region should be associated with graphene layers stacked in parallel^29^. Besides, there are also graphene-like clusters^30^ composed of three to five carbonaceous planes arranged with the spacing close to 0.34 nm. Such spacing, according do PDF 4+ crystallographic database, is typical for 002 direction of graphene. Precise measurement of the spacing was not possible because of misalignments in the amorphous carbon domain.

**Fig. 2:**
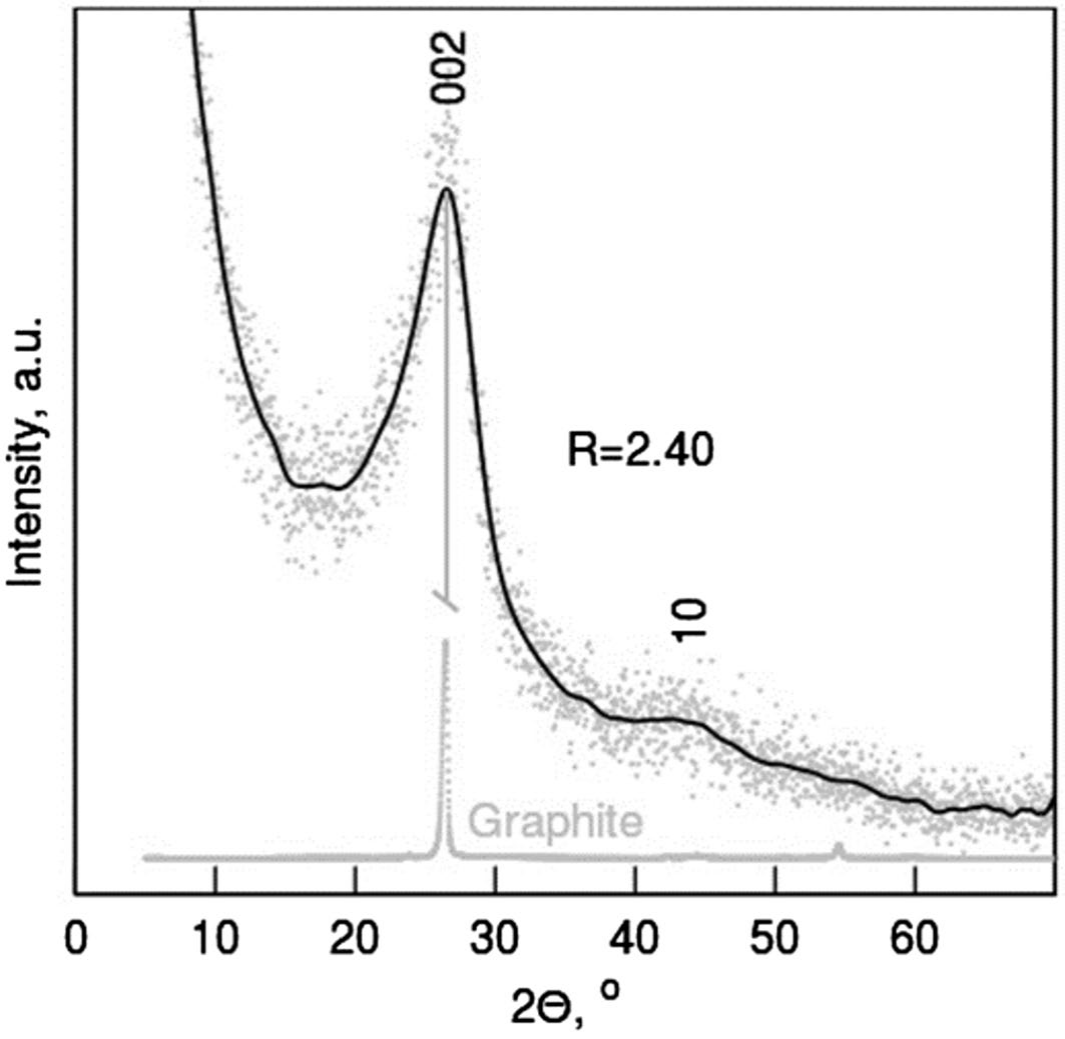
X-ray diffraction patterns of the CH_NaCl sample.

**Fig. 3:**
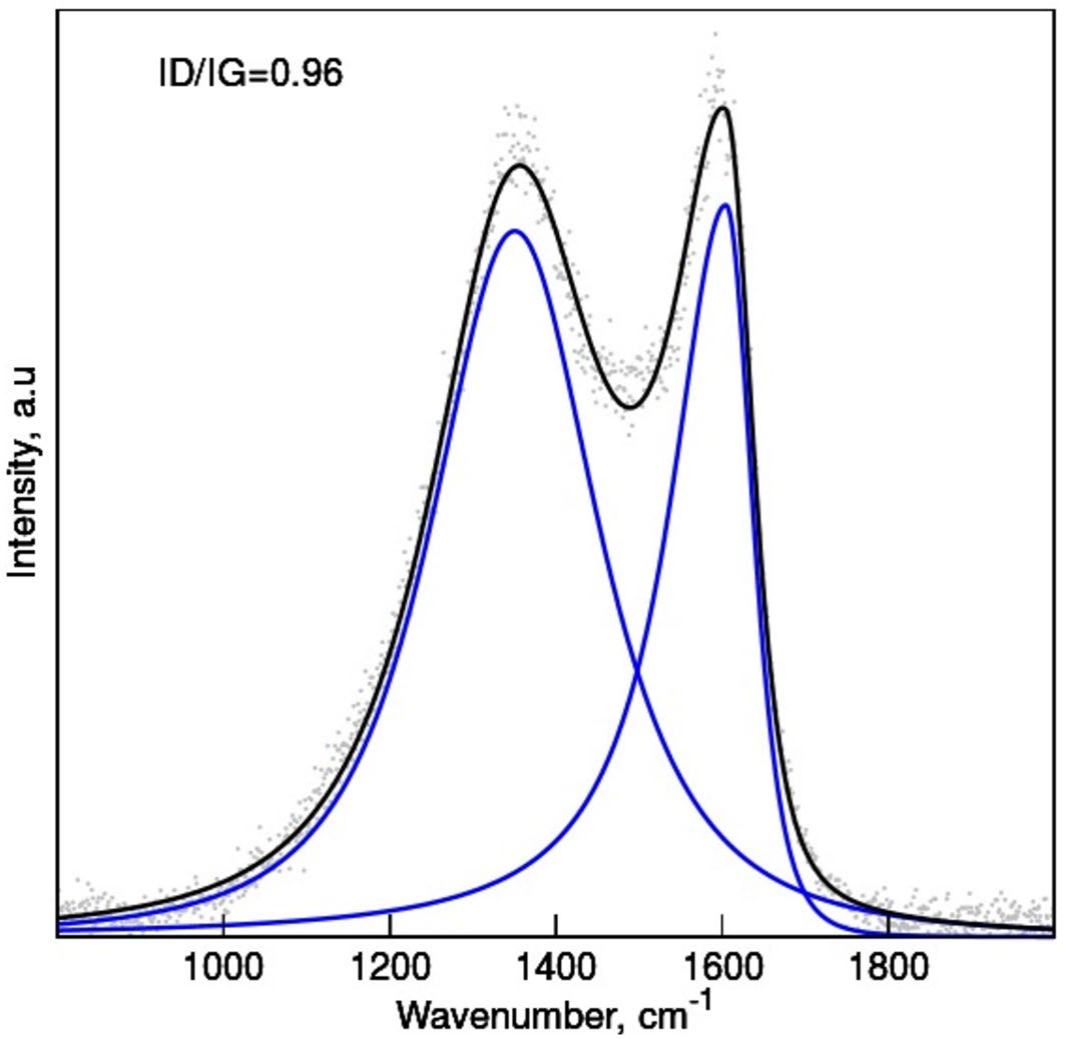
The deconvoluted Raman spectra (blue curves) and raw data (black curves).

Further structurality and crystal phase composition was revealed by XRD (Fig. 2). While regular graphite shows a typical peak at 2θ = 26.0°, corresponding to the interlayer space of 0.335 nm, the structure regularity of the studied samples is represented by a broad peak at ~ 26°, stemming from the (0 0 2) characteristic plane of the turbostratic carbon crystallites^24^. The interlayer separation in these turbostratic graphenic domains (d_002_) was estimated to be 0.34 nm. Similar studies and calculations performed for nanocarbon structures obtained from chitosan pyrolyzed without ZnCl_2_/NaCl salts (CH sample) yielded the interlayer separation of 0.370 nm. Oxygen functionality is seen in the spacing between graphene layers or defects caused by weaker Van der Waals phenomenon^31^. The extent of the crystallites (La) is observed by a peak at ~ 44° with (10) plane. Crystallite height (Lc) coming from lateral extent of stacked graphene layers was calculated from the Scherrer formulas for the X-ray wavelength peak (002). The La, Lc reached 48.9 Å and 19.96 Å, respectively. The mean number of layers was ~ 5. An empirical parameter R was equal 2.40. It represents a measure of carbon sheets set out as a single layer i.e., larger (> 1) values specify a higher amount of graphene sheets stacked in parallel^32^. The turbostratic carbon was further confirmed by Raman spectroscopy (Fig. 3). In graphitic materials the lateral size of the crystallites has been shown to be inversely proportional to the intensity ratio of the D- (~ 1350 cm^−1^) and G-bands (~ 1580 cm^−1^)^33^. The obtained La values are in reasonable agreement with the crystallites dimension estimated in our XRD studies. Both samples exhibit two specific absorptions: D-band and G-band. The D-band displays the disordered sp^3^ hybridized carbon, while G-band shows the turbostratically stacked graphene and indicates the presence of sp^2^ carbon^34^. The results show the level of defects observed for both samples. The ID/IG ratio is larger for N-doped carbon because of the structure defects and edge plane exposure from heterogeneous atom, here nitrogen, incorporated the graphene structure. Heteroatom molecules are also evidenced since the D band reflects the sp^3^ hybridized carbon. Due to the weak intensity, the mode of D + G can be diminished^35^.

**Fig. 4:**
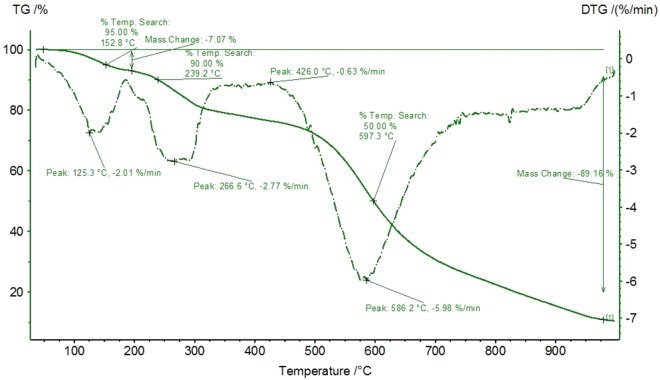
Gravimetric (black curve) and thermal decomposition spectra of the CH_NaCl sample. The dashed line represents heat flow.

The thermal as well as gravimetric behaviour of chitosan mixed with salts (0.42%mol NaCl + 0.58%mol ZnCl_2_) during carbonization in a dynamic nitrogen atmosphere was measured up to 1000 °C (Fig. 4). Melting temperatures of eutectic binary chloride mixtures NaCl + ZnCl_2_ was estimated to 262°C^36^. Individual NaCl has relatively high heat capacities (1.1 kJ kg^−1^ K^−1^) and high melting points (801 °C). Higher amounts of NaCl in the mixture can shield the Lewis acidity of ZnCl_2_ thereby lowering their miscibility^37^. Here, also other parameters like polarity should be addressed. Apart from the fact that both: NaCl and ZnCl_2_ act as the dehydrating agents, they demonstrate the synergistic role of the components and their derivatives like Na_2_ZnCl_4_ which becomes salt melt media at temperature above 300 °C in the used NaCl: ZnCl_2_ ratio. The initial weight loss (20 to 125  °C) is coming from physically adsorbed water’ evaporation. The dehydration, coupled with preliminary deacetylation releases exothermic cross-linking reactions without the chitosan chain degradation up to 300°C^38^. For the sample with salts additional peaks are observed due to the decomposition of ZnCl_2_ at 290 °C. The presence of molten salt shifts the main decomposition of chitosan to the temperatures below 290 °C. Above 400 °C remaining ZnCl_2_ begins to volatilize out of the system^39^. The macroscopic appearance of the monolith hardly changed during further heating up to 586 °C. The results indeed point to the desired salt melt process as a carbonization mechanism showing the high compatibility of the used chitosan with the ionothermal approach. In comparison with our previous studies where the secondary salt was LiCl, no expansion in case of NaCl is observed.

**Fig. 5:**
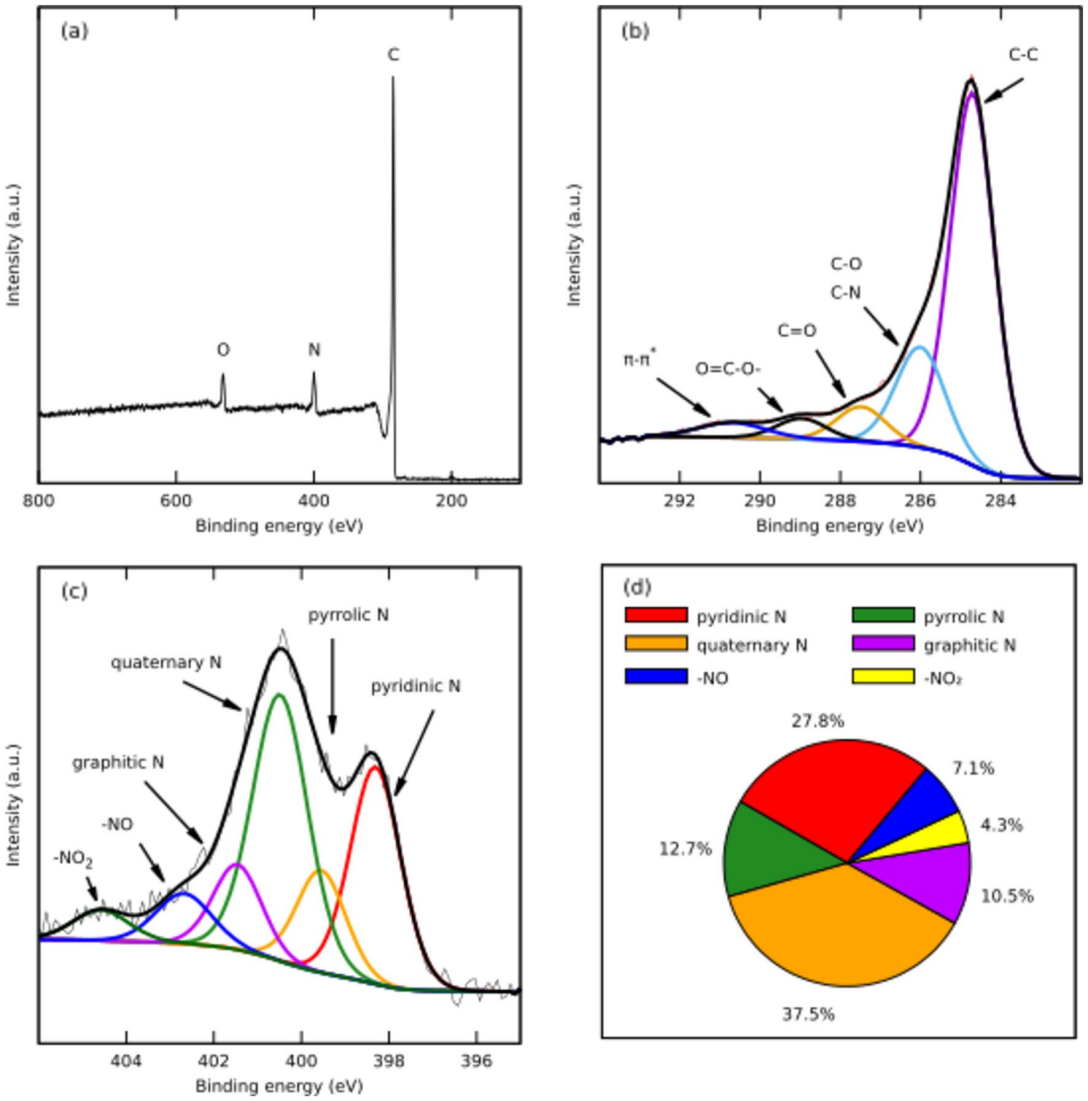
The XPS spectra of the CH_NaCl (**a**) high resolutions spectra; (**b**) the contents of C1s functional groups: (**c**) high resolution XPS spectra of N 1s; (**d**) the overview for all nitrogen functional groups’ content.

The surface individual nitrogen with functional groups was examined using XPS, after deconvolution treatment of the N 1s spectra (Fig. 5) with six peaks (Fig. 5c). The first one refers to pyridinic-N, followed by the pyrrole-like nitrogen. The third and dominant one can be attributed to nonplanar quaternary nitrogen groups. On the basis of XRD results we postulate that due to steric hindrance the incorporation of these groups into the carbon matrix might be restricted to its edges. The graphitic nitrogen and N-oxides of pyridine stem from chitosan^40,41^. The graphitic nitrogen groups possess two pz electrons and have planar configuration. It is not clear whether this p orbital is involved in the 6 π-electron aromatic system or becomes active site due to partial occupation of π* anti-bonding orbitals around nitrogen^42^. As to nonplanar quaternary nitrogen's sites we also postulate their location at the edges of the carbon material due to low interlayer distance (Fig. 5d). Table 1 summarizes the elemental composition of the pyrolized materials, determined by the elemental analysis, confronted with the chitosan precursor. The data show that upon pyrolysis of chitosan, the remaining carbonaceous solid gains on content of carbon and nitrogen.

**Table 1:** Elemental analysis from CHN combustion method.

	Precursor	CH_NaCl [%mas.]
C	38.30	80.00
H	6.70	2.30
N	5.90	8.50
O	49.10	9.20

**Fig. 6:**
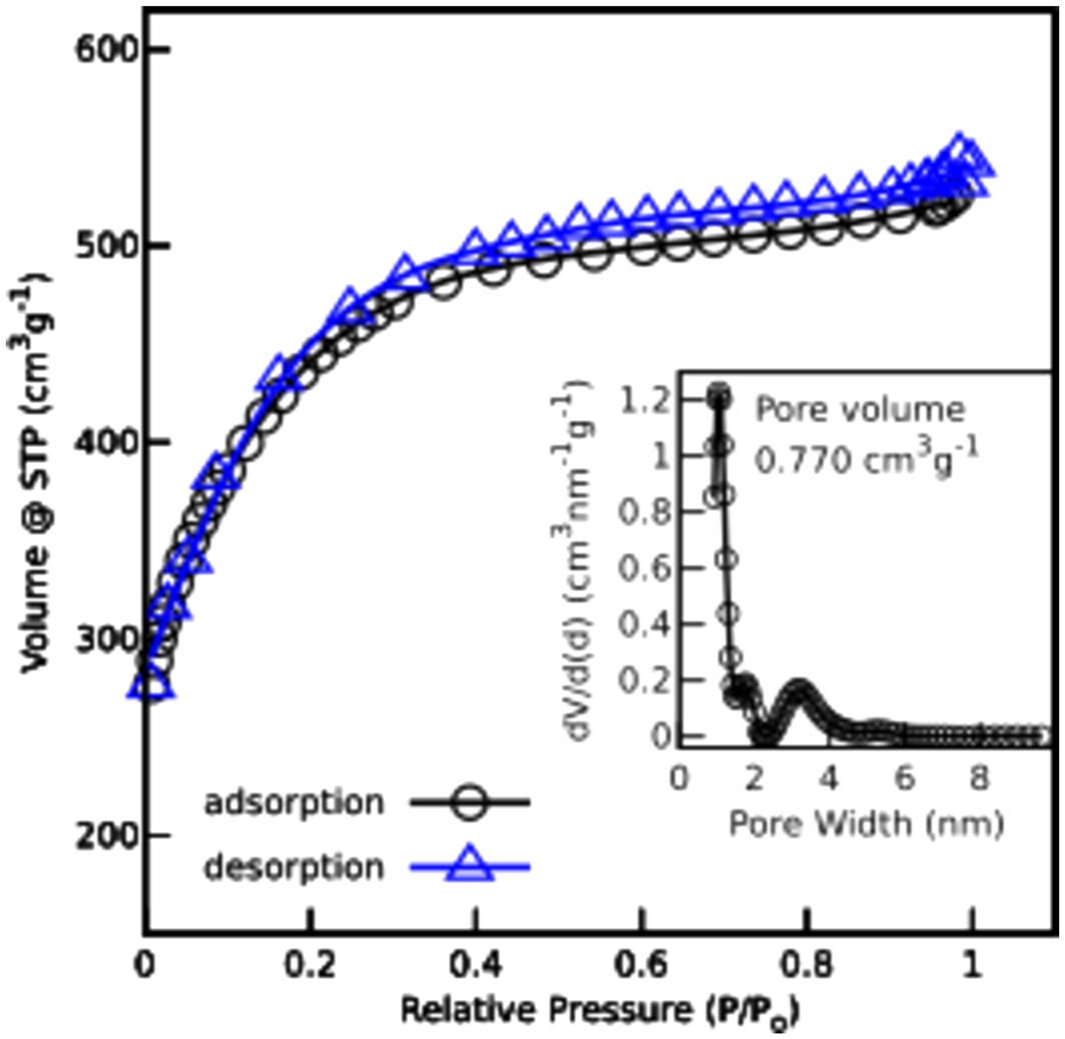
Nitrogen adsorption/desorption isotherms and pore size distributions (inset) of the CH_NaCl sample.

Specific surface area and the pore distribution were assessed by utilizing the data from isotherms. In Fig. 6, the CH_NaCl sample displays the presence of the hysteresis loop (type H4) for different pore sizes from micro- to meso-pores, which further corresponds with the isotherm type of IV. It shows a well-developed hysteresis in the high-pressure region. The adsorption phenomena happen at a low relative pressure (less than 0.2). Further rise of adsorption at 0.2–1.0 P/Po, although at much slower rate, shows the existence of mesopores. As shown in Fig. 6 (inset) the size of the micropores is at ca. 2 nm. Table S1 shows the pores volume, and their specific inner area for the samples synthesized with NaCl. We also quote, for comparison, similar data previously published for the samples obtained with LiCl and without any inorganic salt^43^. Data clearly show, that the presence of NaCl or LiCl during the synthesis favours the formation of pores having large specific volumes and surfaces, but also having much lower average width than the samples produced with no salts. There are also seen some differences between the samples synthesized with NaCl and LiCl. The latter one, holds advantage in almost all areas of the pore's characteristics but their micro size part.

### Electrochemical performance

**Fig. 7:**
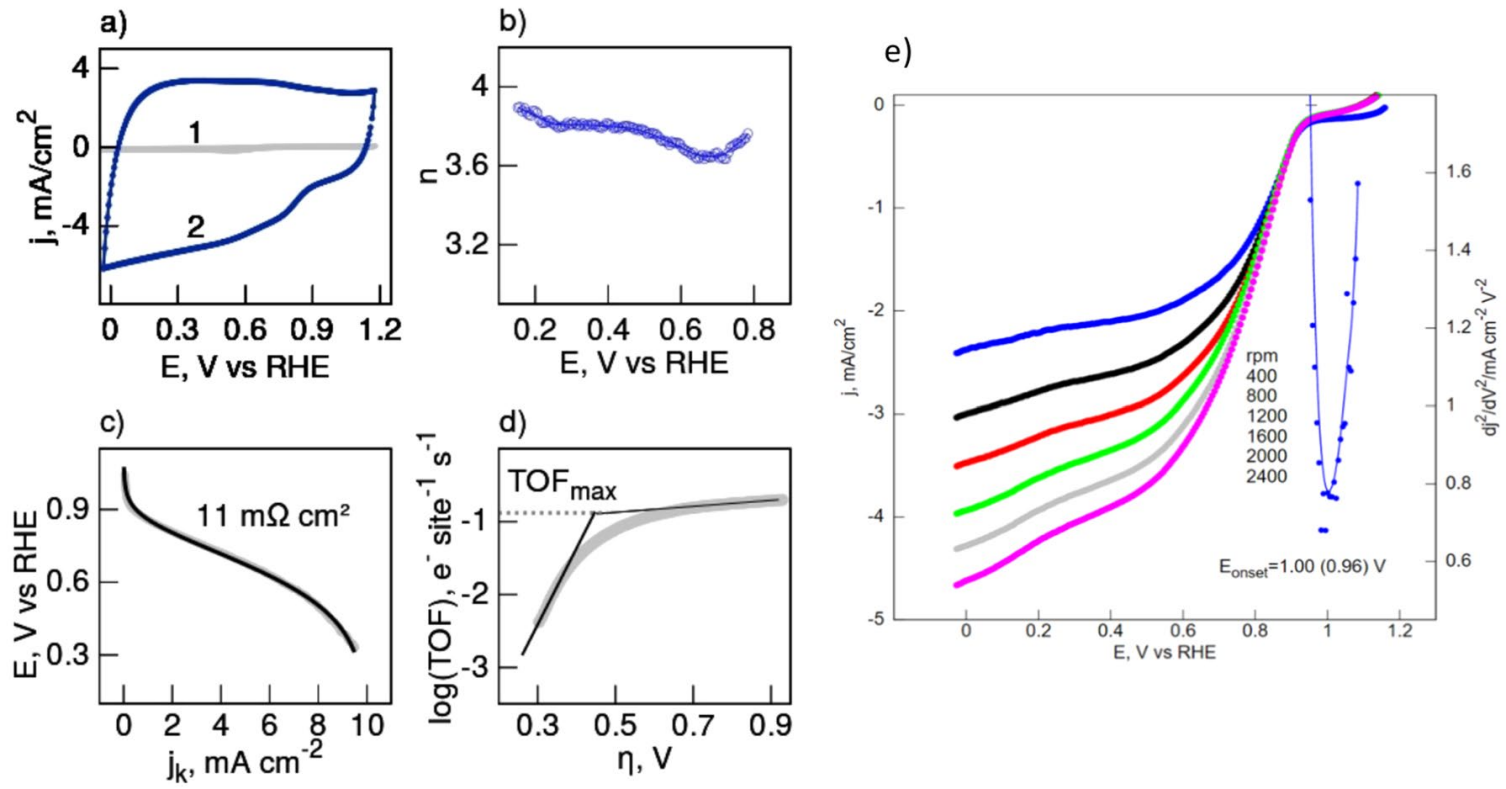
Cyclic voltammograms of CH_NaCl catalyst for the ORR obtained in N_2_ (1) and O_2_ (2) saturated 0.1 M KOH aqueous solution. Scan rate 50 mV/s (**a**); the moles of electrons involved in the ORR calculated from the Koutecky-Levich equation (**b**); polarisation plots of CH_NaCl catalyst for the ORR obtained O_2_ saturated 0.1 M KOH aqueous solution. Simulated curve (black line) yielded ohmic resistivity of the cell (**c**); catalytic Tafel plot derived from Koutecky-Levich treatment of rotating disk data of CH_NaCl catalyst in O_2_ saturated 0.1 M KOH aqueous solution (**d**); linear sweep voltammetry curves of CH_NaCl catalyst for the ORR obtained in oxygen saturated 0.1 M KOH aqueous solution. Scan rate: 10 mV/s. Electrode rotation rates as indicated (**e**).

The ORR catalytic activity of CH_NaCl material towards the O_2_ reduction reaction was studied by the CV measurements (Fig. 7a). When prior to the experiments, the solution was purged with nitrogen, no significant peak or wave were discerned in the voltammogram. On the other hand, in the case of the O_2_-saturated solution a clear cathodic wave turned up at 0.770 V, demonstrating oxygen implication in the electrocatalytic reaction (Fig.S2). The onset potential of the oxygen reduction was estimated by linear sweep voltammetry performed using a rotating disk electrode (Fig. S3). The Koutecky-Levich plots (J^−1^ vs ω1/2) of CH_NaCl catalyst were drawn using data from LSVs for various potentials and all lines show fairly good linearity. The electron-transfer number calculated from the slope of those lines for the electrode potentials from 0.1 to 0.8 V is n = 3.7, on average (Fig. 7b). This parameter was also evaluated for the lithium salt founded carbon (CH_LiCl, n = 3.5) and for the reference carbon obtained by pyrolysis of pure chitosan (CH, n = 2.2) in our previous research^23^.

Figure 7c shows plots of the kinetic currents against potential. The linear regions of the plots allowed, specifically their slopes, estimation of the specific resistivity of the catalyst^44^, which was 11 mΩ cm^2^. This relatively low value could be attributed to the 3D structure of the catalyst, and equal potentials distribution over the catalyst layer. The relevant parameter values reported by us earlier for CH_LiCl and CH were 11 mΩ cm^2^ and 38 mΩ cm^2^, respectively^23^. In order to examine the specific activity of the catalysts the turnover frequency (TOF) for the O_2_ reduction was evaluated. TOF is defined as the number of reduced oxygen molecules per the catalyst active site per second and estimated using the reduction current in the LSV, the XPS and the number of electrons transferred in the reaction. TOF is used here as an indicator of specific activity where the kinetic currents, and molar concentrations of various forms of nitrogen group in a CH_NaCl catalyst is included. TOF is a potential-dependent kinetic parameter that at its maximum value, reached at potentials, where the fraction of active catalyst is near unity, has a defined relationship with homogeneous apparent rate constant (k_obs_)^45^. The Tafel like plots (TOF-η) indicate the potential dependence of TOF in the region of a catalytic voltammogram prior to the current plateau region (Fig. 7d). Table 2 shows data, which allow to correlate TOF with the site concentration of various forms of nitrogen groups in the CH_NaCl catalyst. We also quote data obtained in our previous study for comparison^23^. There is striking discrepancy between results and expectations. The CH_NaCl catalyst has overall lower content of highly active towards ORR pyridinic and pyrrolic sites than that of CH_LiCl, nevertheless it yielded higher catalytic activity in terms of TOF. After stabilizing the cyclic voltammogram curves, we performed LSV in O_2_ saturated 0.1 M KOH electrolyte at different rotating rates. The peak occurs at the same voltage and this is a characteristic of rapid electron transfer kinetics of electrode reactions. The current is measured in response to an applied electrical potential (Fig. 7e).

**Table 2:** The site densities and the turnover frequencies of the N-active sites in the carbon matrix of the studied catalyst (N-oxide groups are excluded).

Sample	S.D./10^20^ (sites/cm^3^)					TOF (e site⁻^1^ s⁻^1^)		Ref
	Pyrydinic	Pyrrolic	Quaternary	Graphitic	Total	Max	0.8 V (RHE)	
CH_NaCl	3.71	1.69	5.01	1.41	11.82	0.132	0.088	This work
CH_LiCl	4.65	2.70	3.27	–	10.61	0.095	0.030	^23^
CH	2.36	0.75	2.99	1.71	7.81	0.045	0.053E-3	^23^

It seems that the catalytic activity of the sites depends not only on their intrinsic chemical properties but also on their concentration on the catalyst surface^46^. CH_NaCl holds advantage over CH_LiCl in terms of the micropores specific volume and area. Thus, the average concentration of the active site on the CH_NaCl surface is larger than that of CH_LiCl. Indeed, a creation of appropriate microporosity in N-doped carbon is immensely important for a particular electrochemical reaction^47–51^. A molten-salt-mediated synthesis of porous N-doped carbon can lead to the efficient cathode catalysts for advanced sources like microbial fuel cells^52^ or low temperatures fuel cells^53^. The carbon material described by us in substituting LiCl/NaCl by KCl was reasonably good as a supercapacitor but performed very badly in the electroreduction of oxygen studies^54^.

## Conclusions

In summary, we have demonstrated a synthetic path to produce the highly microporous carbonaceous catalyst from chitosan pyrolyzed in the molten eutectic salt mixture of ZnCl_2_ and NaCl. The catalyst exhibited catalytic activity towards ORR via a direct four-electron pathway. It holds advantage over sample pre-treated with LiCl in terms of the micropores specific volume and area, seen as their percentage rate, measured in the BET. Thus, the average concentration of the active site on the surface is larger. The current report clearly shows that carbons obtained with the assistance of sodium salts perform (towards ORR) almost as good as the samples produced with lithium salts. Their practical advantages boils down to wider availability and lower price of NaCl. The appearance of NaCl as a salt melt is sustainable as this is a common salt which could reduce the cost of the process owing to the fact that it could be recovered from a cheap sources.

This catalytic activity comes from the relatively high concentration of the pyridinic and pyrrolic groups in the bulk of the catalyst, and could be also related to their large surface concentration, especially in the micropores. It seems that the catalytic activity of the sites depends not only on their intrinsic chemical properties but also on their concentration on the catalyst surface. Indeed, a creation of appropriate microporosity in N-doped carbon is immensely important for a particular electrochemical reaction. A molten-salt-mediated synthesis of porous N-doped carbon can lead to the efficient cathode catalysts for advanced power sources. The further research planned to be carried out will concern the detailed determination of properties and relations for a specific application i.e. Zn-air batteries.

## Experimental section

### Materials

- Chitosan, medium molecular weight and deacetylation degree 81% (Sigma Aldrich)
- anhydrous Sodium chloride (Sigma Aldrich)
- anhydrous Zinc chloride (Sigma Aldrich)

All reagents were bought from vendors and used without purification.

### Methods

The chitosan (3 g) was exhaustively blended with the soluble eutectic salts (3.51 g of ZnCl_2_ and 0.99 g of LiCl) just before the pyrolysis up to 800 °C under nitrogen atmosphere with a heating rate of 3 °C min⁻^1^. After then, 3 M HCl removed any inorganic matter from the carbon by the orbital shaker and further sonication treatment, and filtration till the filtrate reached the neutral pH value. The washed carbons (CH_NaCl) were dried under vacuum conditions for 24 h at 80 °C. Pyrolysed pure CS serves as a reference used here (CH)^23^.

### Physical characterization

Thermal analysis was captured by Thermal Gravimetric Analysis (TGA) from NETZSCH TG 209F3 TGA209F3A-0346-L. The measurements were conducted in the temperature values of 40–1000 °C, with a heating rate of 3 °C min⁻^1^ under N_2_. The Raman Scattering Spectroscopy with Renishaw 2000 system (excitation by diode laser 514 nm, laser power of 25 mW) was operated in backscattering geometry. X-ray photoelectron spectroscopy (XPS) study was assigned for analysis of the surface functional groups using the VG Scienta250Xi spectrometer (Prevac Sp. z o.o.) with Al Ka radiation (hν = 1486.6 eV). The binding energy scale setting the C 1s transition at 284.6 eV. XRD patterns were retrieved using Siemens D5000 X-Ray Powder diffractometer (Cu radiation) with a step size of 0.03**°.** The surface properties were determined by the Brunauer–Emmett–Teller (BET) method and quenched solid density functional theory on the Quantachrome NovaWin (Version 11.03). The CHN Analyser 2400 (Perkin-Elmer, Germany) provides elemental composition by CHN combustion method.

### Electrochemical analysis

The electrochemical measurements (cyclic voltammetry CV and linear sweep voltammetry LSV) were conducted in 0.1 M KOH, in a three-electrode configuration provided by Autolab PGSTAT 101, with a glassy carbon (GC) electrode used as a working electrode. An Ag/AgCl electrode (reference) with saturated KCl aqueous solution and a platinum wire (counter electrode) were applied. All potentials in this report were converted into reversible hydrogen electrode (RHE) scale by adding 0.965 V. The 'real' current from ORR, the LSV measurements were performed in both N_2_ and O_2_, and then removed the current of N_2_ from that of O_2_ in order to get rid of the significant the double-layer capacitance seen in carbons. 1.5 mg of catalyst was blended with 50 μL of Nafion, 50 μL of isopropanol and 900 μL of deionized water, followed by ultrasonic treatment for 30 min. From the slurry, 5 μL droplet was casted onto the electrode area (diameter = 3 mm) and naturally dried in air. CV was performed with a scan rate of 50 mV s^−1^ from 0.2 V to −1.2 V. LSV was accomplished with a scan rate at 10 mV s^−1^ from 0.2 V to −1.2 V (400, 800, 1200, 1600, 2000 and 2400 rpm). All procedures and protocols were accomplished as in our previous works^23^. The shift in onset potential as well as overpotential at a defined current density of 10 mA cm^−2^ is usually used as a benchmark current density (indicative parameter of electrocatalyst).

## Supplementary Information

Supplementary Information.

## Data Availability

All data generated or analysed during this study are included in this published article (and its Supplementary Information files). Raw experimental data, including raw diffraction data, are available at https://doi.org/10.5281/zenodo.8265309.
