# Supplementary material for: Chitosan pyrolysis in the presence of a ZnCl2/NaCl salts for carbons with electrocatalytic activity in oxygen reduction reaction in alkaline solutions
Source: Sci Rep. 2024 Oct 8;14:23374. doi: 10.1038/s41598-024-72411-1 (PMC11461666; doi:10.1038/s41598-024-72411-1)
Supplement: Supplementary file 1 — Supplementary Information. [file 41598_2024_72411_MOESM1_ESM.pdf]

Chitosan pyrolysis in the presence of a $ZnCl_2$/NaCl salts for carbons with electrocatalytic activity in oxygen reduction reaction in alkaline solutions.

Maria K. Kochaniec[a] and Marek Lieder[b]*

[a]Faculty of Chemistry, Warsaw University of Technology, Noakowskiego 3, 00−664 Warsaw, Poland, e-mail: maria.kochaniec@pw.edu.pl

[b]Chemical Faculty, Department of Process Engineering and Chemical Technology, Gdansk University of Technology, Narutowicza 11/12, 80−233 Gdansk, Poland

Supplementary information

1. The samples were characterized by scanning electron microscopy FEI Quanta 3D ESEM/FIB.

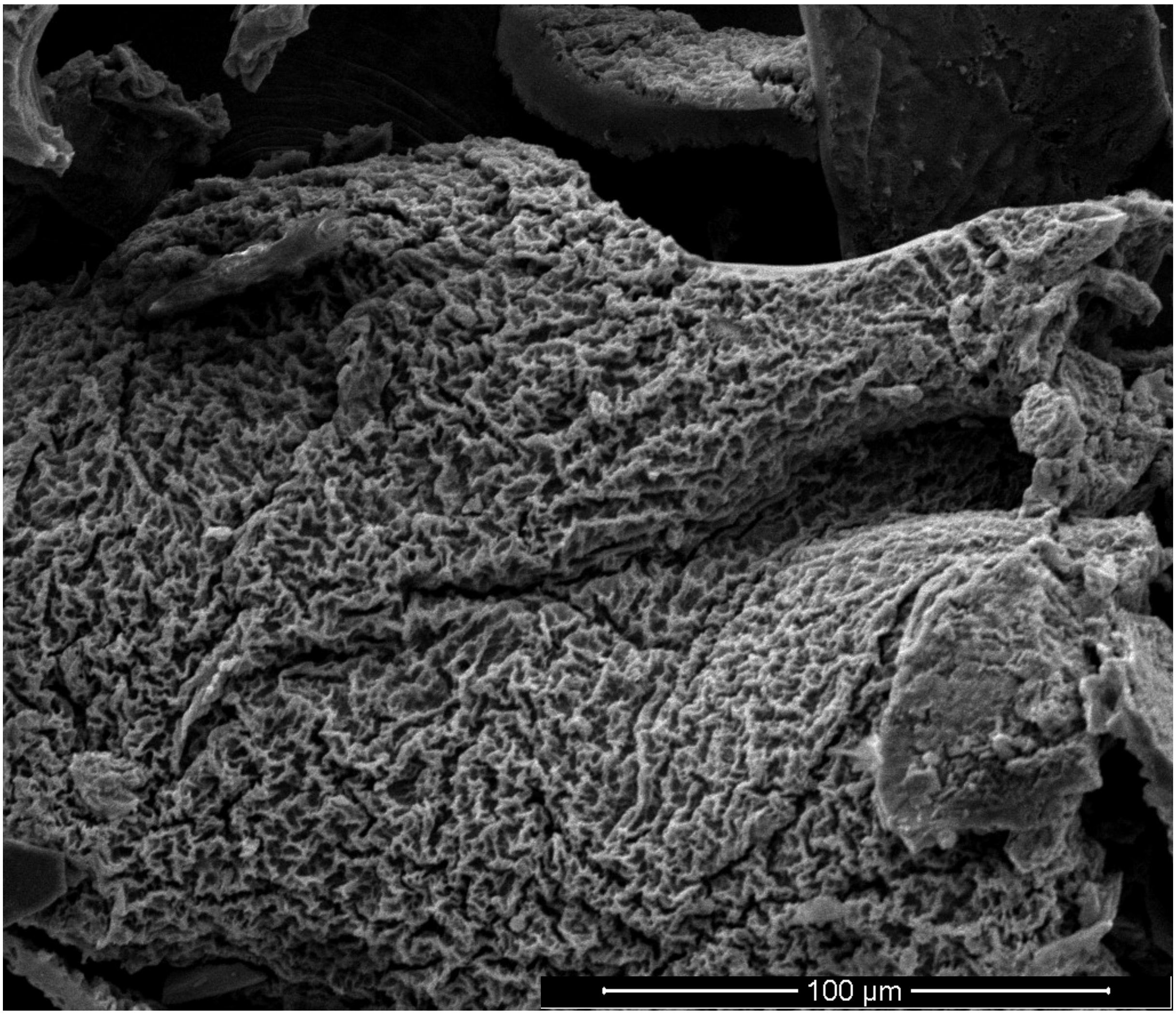

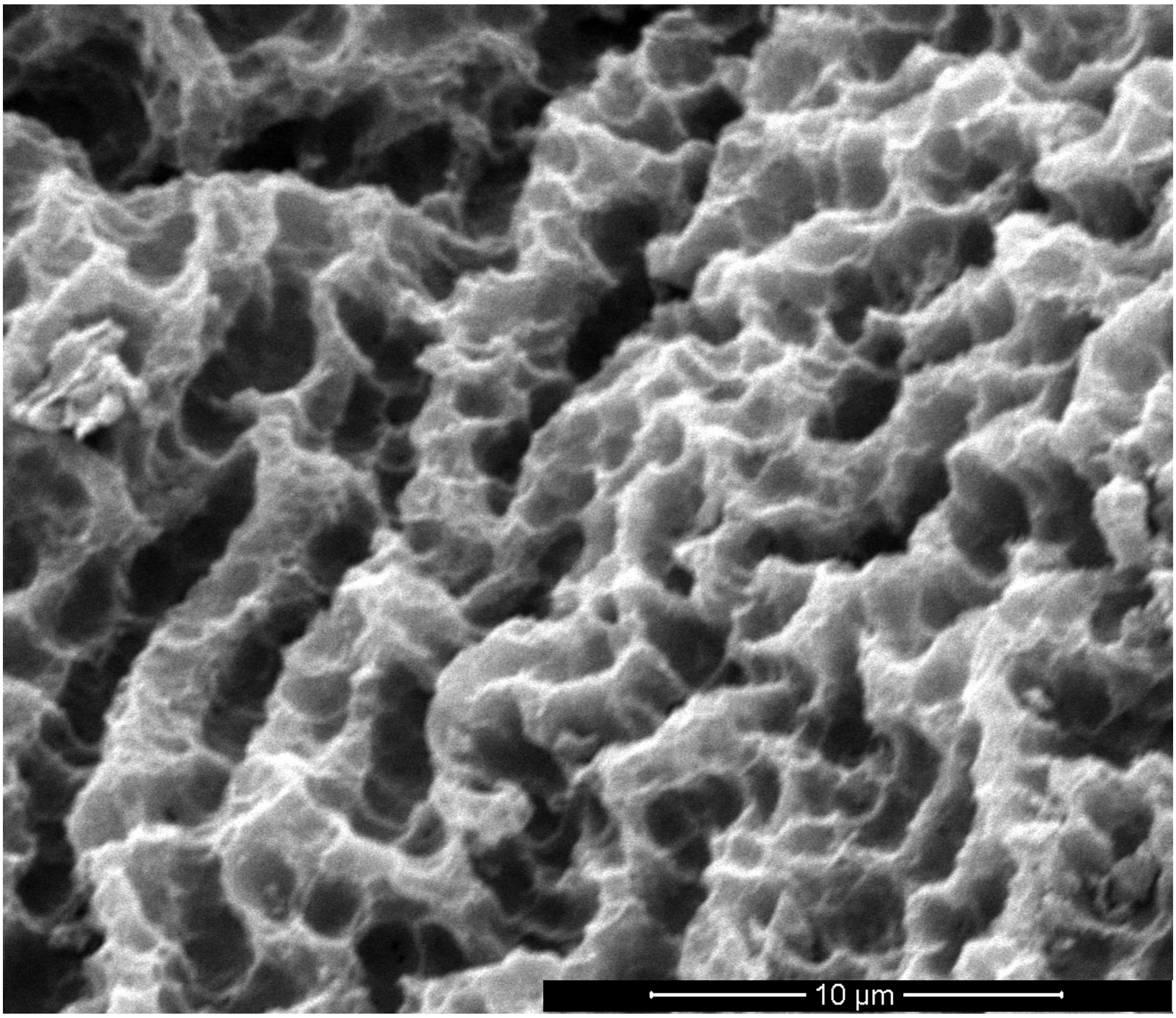
10 µm

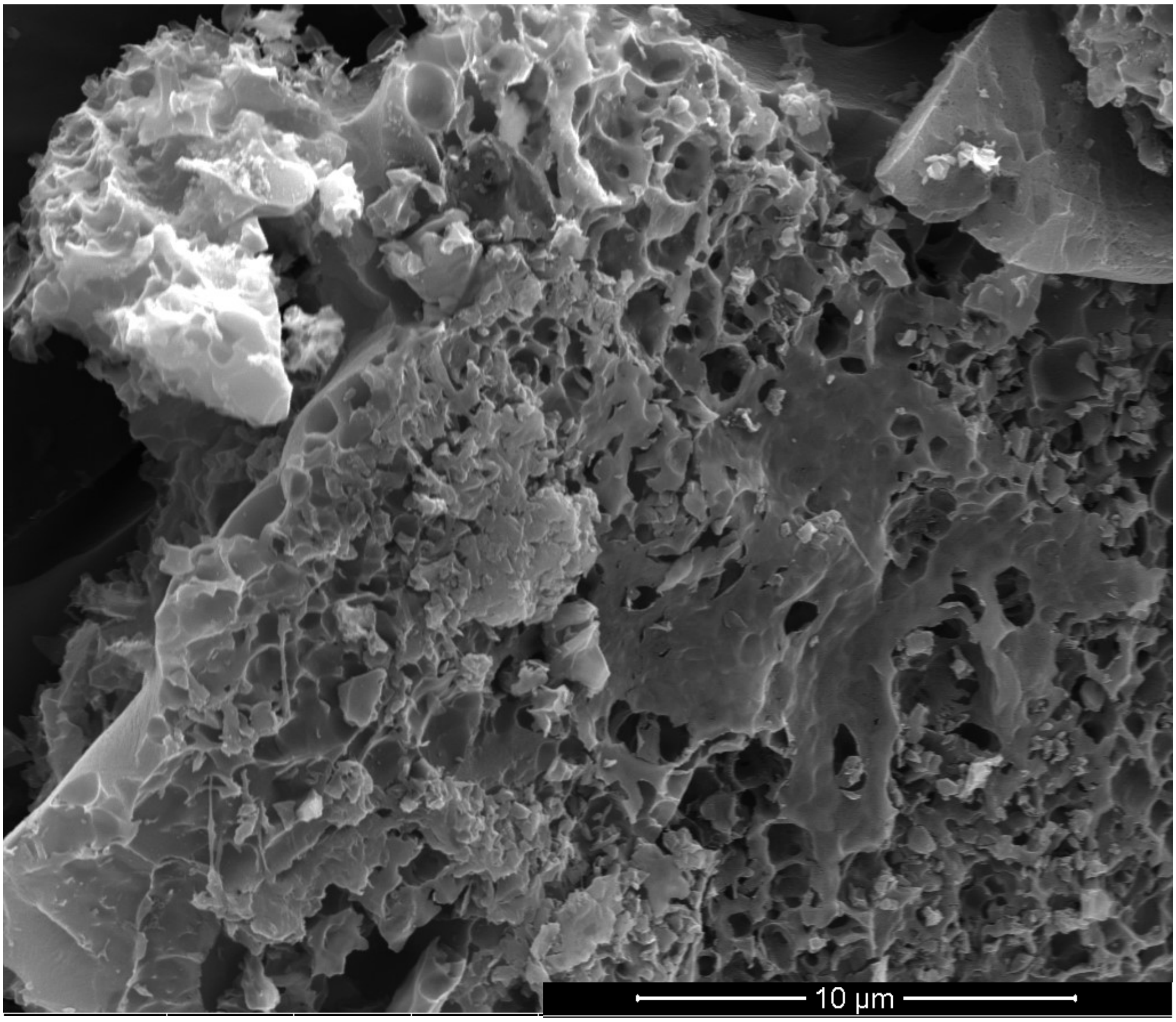

Fig. S1. SEM images of the CH_NaCl samples

2. All potentials were converted into reversible hydrogen electrode (RHE) scale by adding 0.965 V. The 'real' current from ORR, the LSV measurements were performed in both N_2 and O_2, and then removed the current of N_2 from that of O_2 in order to get rid of the significant the double-layer capacitance seen in carbons.

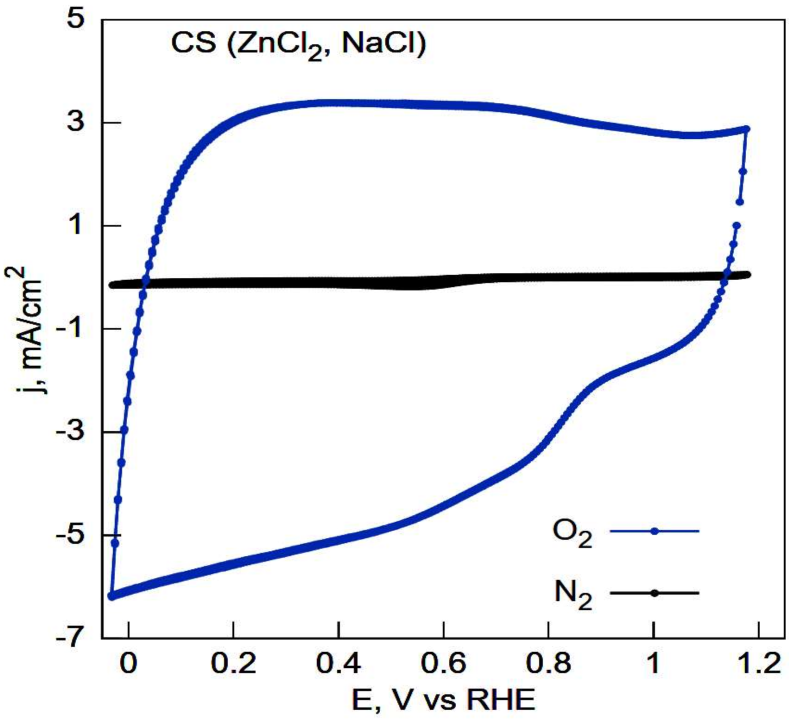

Fig. S2. Cyclic voltammograms of the CH_NaCl samples in saturated 0.1M KOH solution

a

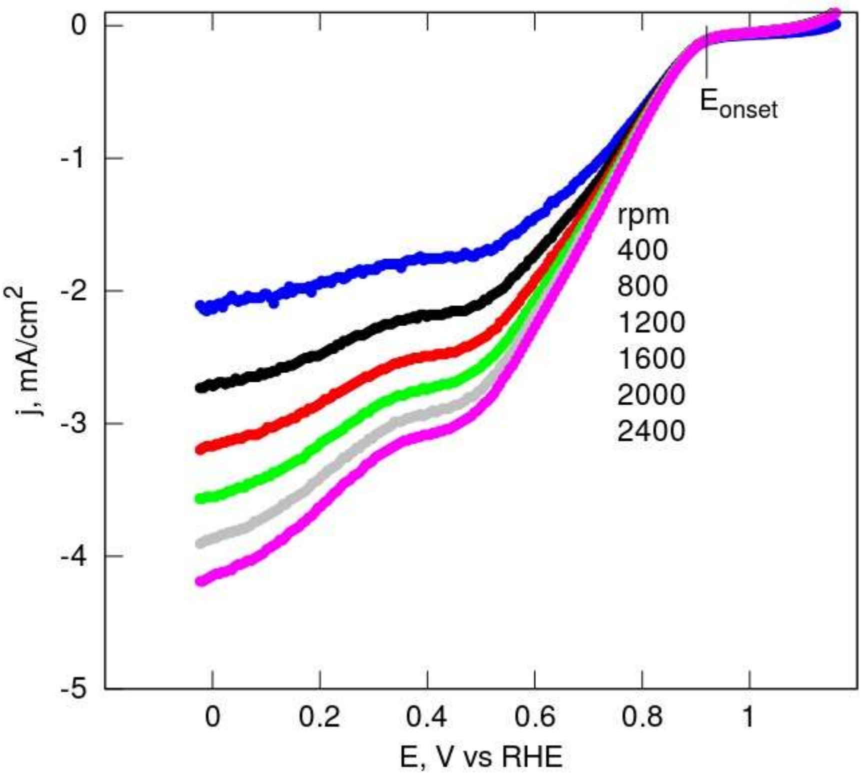

b

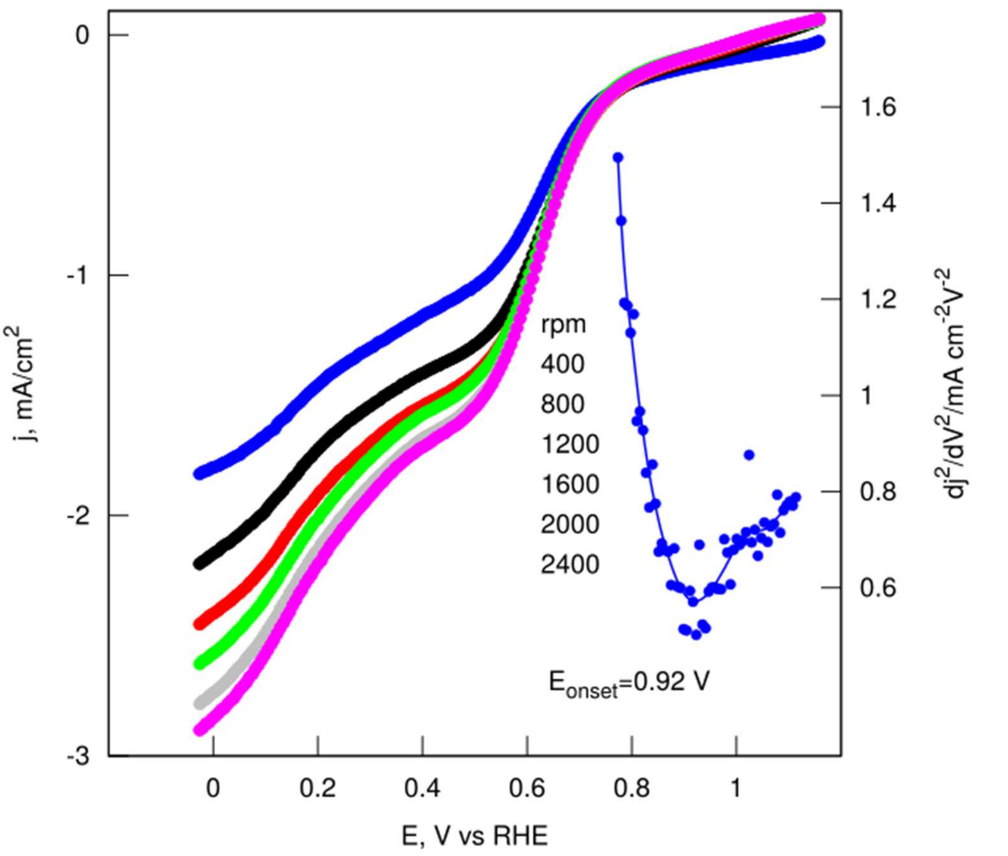

Fig. S3. Linear sweep voltammetry curves (a) of CH_LiCl catalyst for the ORR obtained in oxygen saturated 0.1 M KOH aqueous solution; Linear sweep voltammetry curves of CH_NaCl catalyst for the ORR obtained in oxygen saturated 0.1 M KOH aqueous solution (b). Scan rate: 10 mV/s. Electrode rotation rates as indicated. Fig. S3 (a) Rybarczyk, M. K., Gontarek, E., Lieder, M. & Titirici, M. M. Salt melt synthesis of curved nitrogen-doped carbon nanostructures: ORR kinetics boost. Appl. Surf. Sci. (2018) doi:10.1016/j.apsusc.2017.11.064 with permission from Elsevier.

The number of electrons was calculated from the current with the angular velocity of the disk ω using Koutecky–Levich (K-L) plots of CH_NaCl catalyst for the ORR obtained in O2 saturated 0.1 M KOH at selected potentials (from 400 rpm to 2400 rpm). From all the date we got the current density and corresponding rpm value. From the plot J^{-1} vs $\omega^{-1/2}$, we got the K-L plots. Finally, from the slope of the line we got the number of electron transfer in ORR. It was determined by the K–L equation:

$$\frac{1}{j} = \frac{1}{j_k} + \frac{1}{j_i} = \frac{1}{nFAkC} + \frac{1}{0.62nFAD^{\frac{2}{3}}\omega^{\frac{1}{2}}v^{-\frac{1}{6}}C}$$

where *j* represents the measured current density, *jk* and *jl* are the kinetic and the diffusion-limited current densities, respectively. The electron transfer in the oxygen reaction is described by *n*, *F* is the Faraday constant (96485 C/mol), *A* is the electrode area, *k* is the rate constant for the oxygen reduction, *D* is the diffusion coefficient of oxygen in the electrolyte (1.9×10^{-5} cm^2/s), *ω* is the angular velocity of the electrode, *v* is the kinematic velocity of the electrolyte (0.01 cm^2/s) and *C* is the concentration of saturated oxygen in the electrolyte

(1.2×10^{-3} mol/L). The number of electrons n transferred during the oxygen reduction can then be calculated by the slope, when plotting $1/j$ vs. $1/\omega^{1/2}$.

TOF was calculated based on the formula:

$$\text{TOF [Turn Over Frequency]} = \frac{\text{current [A/cm}^2\text{] [C/s]}}{1.6\text{x}10\text{^-}19 \text{ x S.D. x } \tau} \quad [1/s]$$

S.D. - volumetric site density [sites/cm³]

τ - thickness of the layer [cm]

Table S1. Pores surface and volume of carbonaceous samples obtained by CS pyrolysis.

Sample	Total area $m^2\ g^{-1}$	Micropore area $m^2\ g^{-1}$	Mesopore area $m^2\ g^{-1}$	Total pore volume $cm^3\ g^{-1}$	Micro-pore vo-lume $cm^3\ g^{-1}$	Meso-pore vo-lume $cm^3\ g^{-1}$	Pore With	Ref
CH_NaCl	1217	948	249	0.770	0.539	0.231	1.051	This work
CH_LiCl	1318	709	610	1.230	0.380	0.850	2.600	[23]
CH	7.8	n/a	n/a	0.02	n/a	n/a	3.939	[23]
